# Assessment and comparison of model estimated and directly observed weather data for prediction of diarrhoea aetiology

**DOI:** 10.1017/S0950268824001183

**Published:** 2024-10-09

**Authors:** Ben J. Brintz, Josh M. Colston, Sharia M. Ahmed, Dennis L. Chao, Benjamin F. Zaitchik, Daniel T. Leung

**Affiliations:** 1 Division of Epidemiology, University of Utah School of Medicine, Salt Lake City, UT, USA; 2 Division of Infectious Diseases and International Health, University of Virginia School of Medicine, Charlottesville, VA, USA; 3 Division of Infectious Diseases, University of Utah School of Medicine, Salt Lake City, UT, USA; 4 Institute for Disease Modeling, Bill & Melinda Gates Foundation, Seattle, WA, USA; 5 Department of Earth and Planetary Sciences, Johns Hopkins University, Baltimore, MD, USA

**Keywords:** Clinical Prediction, Infectious Diarrhoea, Weather, Aetiology, Global Health

## Abstract

Recent advances in clinical prediction for diarrhoeal aetiology in low- and middle-income countries have revealed that the addition of weather data to clinical data improves predictive performance. However, the optimal source of weather data remains unclear. We aim to compare the use of model estimated satellite- and ground-based observational data with weather station directly observed data for the prediction of aetiology of diarrhoea. We used clinical and etiological data from a large multi-centre study of children with moderate to severe diarrhoea cases to compare their predictive performances. We show that the two sources of weather conditions perform similarly in most locations. We conclude that while model estimated data is a viable, scalable tool for public health interventions and disease prediction, given its ease of access, directly observed weather station data is likely adequate for the prediction of diarrhoeal aetiology in children in low- and middle-income countries.

Infectious diarrhoea is a significant public health concern, particularly in low-resource settings where access to clean water and sanitation is limited. The incidence of infectious diarrhoea is influenced by a variety of factors, including seasonality and weather conditions [1]. Previous studies have shown that weather conditions, such as precipitation, are strongly associated with the incidence of infectious diarrhoea in low and middle-income countries (LMICs) [2, 3]. Weather conditions in the months prior to illness have also been found to be predictive of the incidence of specific causes of diarrhoeal illness, such as rotavirus [4] and Shigella [5]. In addition, we have previously shown that directly observed (DO) data from weather stations accessed through the Global Surface Summary of the Day online repository can be used to derive the local season [6] or a moving average of recent weather conditions [7] and incorporated with clinical variables to improve prediction of the aetiology of infectious diarrhoea in children living in LMICs. These models can then be adapted to clinical decision-support tools for improved stewardship of antimicrobial and diagnostic use [8]. Gridded meteorological estimates from models using satellite- and ground-based observational data, such as that obtained from the Global Land Data Assimilation System (GLDAS), enable access to granular temperature and precipitation data over time and space that have no missing data, unlike the DO weather station data. In this analysis, we use data from a large multi-centre case–control study of children with moderate to severe diarrhoea to assess and compare the performance of the model estimated (ME) data of GLDAS to the DO weather station data of the Global Historical Climatology Network daily (GHCNd) when making predictions of individual-level diarrhoeal episode aetiology.

We tested the predictive performance of the weather data applied to the Global Enteric Multicenter Study (GEMS), which we previously used to derive and test prediction models for a viral-only aetiology versus other aetiologies of diarrhoea [6, 7, 9, 10]. Briefly, the GEMS was an observational case–control study conducted between 2007 and 2011 at healthcare facilities in 7 countries, in which 9 439 children with moderate-to-severe diarrhoea were enrolled at local healthcare centres along with one to three matched community control-children. A faecal sample was taken from each child at enrollment to identify enteropathogens and clinical information was collected. We used the quantitative real-time PCR-based (qPCR) attribution models developed by [11] in order to best characterize the cause of diarrhoea. Using, only diarrhoea cases, we defined viral aetiology as a diarrhoea episode with at least one viral pathogen with an episode-specific attributable fraction (AFe ≥ 0.5) and no bacterial or parasitic pathogens with an episode-specific attributable fraction. Other aetiology includes any episode with at least one bacterial or parasite pathogen with an episode-specific attributable fraction. Prediction of viral attribution is clinically meaningful since it indicates that a patient would not benefit from antibiotic therapy.

We used daily aggregates of gridded meteorological estimates extracted from GLDAS, which includes 3-hourly weather information on a 25 × 25 km grid, based on the locations of GEMS hospital for each country [2, 12]. The temperature is extracted as minimum and maximum daily temperature. We averaged the daily minimum and maximum temperature in order to calculate an average daily temperature. Although, the average of the min and max is not necessarily equivalent to the daily average, prior literature has shown agreement between this approach and various other approaches used to get a daily temperature summary as well as ground-based measures [13]. We additionally extracted the total daily precipitation in millimetres.

We used the GHCNd to obtain DO average daily temperature and daily total precipitation. The GHCNd combines daily observations from over 30 different sources of climate observations, and undergoes a quality assurance process approximately weekly [14]. We utilized the *readr* package in R to directly download station data from GHCN daily during the study period. For each GEMS study site, we selected the closest weather station, based on haversine distance, that contained data during the GEMS years. For each location, missing weather data was completed using the most recent non-missing weather data prior to it from that same station. We note that there were only missing precipitation values and that missing values only followed days with no precipitation. The max number of missing precipitation values was 173 in Mali, of which the dates coincided with the dry season starting in late October.

We used logistic regression to assess the predictive performance of weather variables on viral versus other aetiology. The term 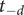 refers to 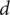 days prior to the day of the patient visit to the health centre. We fit a separate model using the average exposures over each of the following moving windows of exposure:

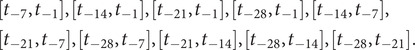

Each model contained three exposure covariates: (1) temperature, (2) precipitation, and (3) their interaction.

We assessed the predictive performance of the model fits with various performance metrics using repeated cross-validation. We conducted Monte Carlo cross-validation by generating 500 random 80% training and 20% testing splits and compared model performance between GLDAS data and GHCNd data using the models with covariates calculated using the intervals described above [15]. Within each iteration, we calculated the area under the receiver operating characteristic curve (AUC), the calibration intercept and slope of the model for assessing weak calibration [16], the area under the precision-recall curve (PRAUC), and we used bootstrapping to compare the AUC between models [17]. That is, bootstrapped differences between two models’ AUCs were calculated, standardized, and compared to the normal distribution. The PRAUC, though less common than the AUC, can be more pertinent when assessing the performance of predictive models for outcomes with imbalanced classes due to its emphasis on the performance when the ground truth is the positive class. In this case, viral etiologies account for approximately 1/3 of the outcomes.

With this approach, we assessed whether certain intervals of weather conditions prior to the presentation of the patient are more predictive of viral aetiology than others, including some intervals which do not start immediately prior to the day of presentation. Finally, we assessed the performance of cross-validated predictions by site. We ordered them by distance to the reporting weather station to assess whether there was a relationship between distance and the relative average AUC from models using the different data sources.

We found that when we plotted the ME and DO temperatures and the precipitation in time series by site, they were very similar. However, the Modelled estimates outperformed the directly observed data in both AUC and PRAUC at each interval explored with the best AUC from each source being 0.66 and 0.64, and the best PRAUC from each source being 0.47 and 0.44, respectively (Figure 1). The best-performing models used the averaged exposure over moving windows which include the most recent days from the health care facility visit.

**Figure 1.**
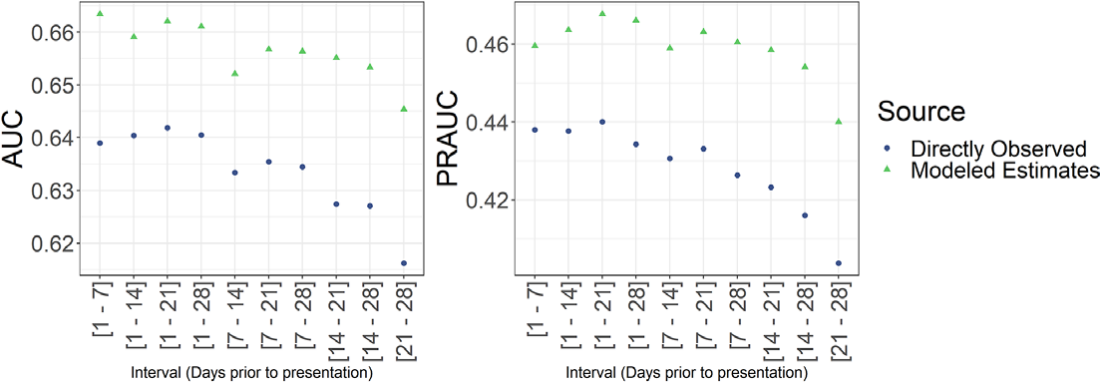
Average cross-validated AUC and PRAUC for model estimated and directly observed weather data averaged exposure over various moving windows.

When predictions from cross-validation were broken down by site, the superior predictive performance using ME over DO data was limited to The Gambia with a difference in average AUC, that is, about 0.102, or about 10%. Notably, the Gambia’s clinical study site is 77 km away from the closest weather station, the fourth farthest site from its weather station. The next largest difference in average AUC between data sources by site in the 1–7 days interval was 0.038 in Mali. When we used bootstrapping within each iteration of cross-validation to compare AUCs between models driven by the two sources of data using the 1–7 days interval for all sites, we found that the median *p*-value in cross-validation is 0.025. This suggests that there was evidence of a difference between models fit using the two sources of data; however, when we conducted the same bootstrapping test and compared the two sources of data within sites, Gambia had a median *p*-value of 0.055, while the other sites had median *p*-values of 0.291 and above. We additionally found that all models satisfied the criteria for weak calibration, that is, on average, the models do not over- or underestimate risk and do not give overly extreme or modest risk esimates. We estimated the calibration intercepts were close to 0 with 95% CIs that contain 0 and the calibration slopes were close to 1 with 95% CIs that contain 1, satisfying the criteria.

Clinicians in low-resource settings are often required to make clinical decisions of infectious syndromes without information from laboratory diagnostics. Traditional clinical prediction rules typically focus on data from the patient at presentation, but more recently, the use of location-specific (or patient-extrinsic) data sources such as climate have shown to improve the performance of prediction rules over clinical factors alone [6, 7]. The optimal method for estimating climate and weather data for incorporation into clinical prediction rules has not been assessed. We demonstrated that on average, while DO weather station data vary in availability, there is ultimately a similar predictive performance as the ME weather data based on satellite- and ground-based observation, for the prediction of aetiology of diarrhoea in paediatric patients.

In both methods, though most prominently with ME weather, we found that the top 4 models by AUC all included temperature and precipitation data with the shortest lag length, (i.e., included the prior days’ weather data in the aggregate predictors), consistent with the known short incubation periods of most enteropathogens, and previous findings observed with individual organisms [2]. The top 3 models by PRAUC also included the shortest lag.

We note some discrepancies in data preparation between the two sources of data. First, the DO data were less complete than the ME, for example, precipitation missing in periods of time where there was no precipitation. In contrast, by its nature, the model estimates had no missing data. However, when using this estimated data, advanced data storage and data cleaning will likely be required for any given application due to its granularity in raw form. Although we found evidence of an average difference between predictive performance, this difference can be attributed to differences at a single site (The Gambia). We did not find a clear linear trend in performance difference when regressed on distance from closest weather station (*p*-value = 0.66, Pearson correlation = 0.2). Further explorations should be made to assess if the best-performing lags from the two sources of weather data apply to other prediction outcomes, and whether our findings can be generalized to other climate factors such as humidity or environmental factors such as air pollution.

In conclusion, we found that GHCNd’s DO weather station-derived data is likely adequate for the prediction of diarrhoeal aetiology in children in LMICs.

## Data Availability

R code and analytic data files are available at https://github.com/LeungLab/CompareMEandDOweather.
